# **α**Synuclein and Mitochondrial Dysfunction: A Pathogenic Partnership in Parkinson's Disease?

**DOI:** 10.1155/2012/829207

**Published:** 2012-06-10

**Authors:** David Protter, Charmaine Lang, Antony A. Cooper

**Affiliations:** ^1^Diabetes and Obesity Program, Garvan Institute of Medical Research, Sydney, NSW 2010, Australia; ^2^School of Biotechnology and Biomolecular Sciences, University of New South Wales, Sydney, NSW 2052, Australia

## Abstract

Parkinson's Disease (PD) is a complex, chronic, progressive, and debilitating neurodegenerative disorder. Neither a cure nor effective long-term therapy exist and the lack of knowledge of the molecular mechanisms responsible for PD development is a major impediment to therapeutic advances. The protein *α*Synuclein is a central component in PD pathogenesis yet its cellular targets and mechanism of toxicity remains unknown. Mitochondrial dysfunction is also a common theme in PD patients and this review explores the strong possibility that *α*Synuclein and mitochondrial dysfunction have an inter-relationship responsible for underlying the disease pathology. Amplifying cycles of mitochondrial dysfunction and *α*Synuclein toxicity can be envisaged, with either being the disease-initiating factor yet acting together during disease progression. Multiple potential mechanisms exist in which mitochondrial dysfunction and *α*Synuclein could interact to exacerbate their neurodegenerative properties. Candidates discussed within this review include autophagy, mitophagy, mitochondrial dynamics/fusion/fission, oxidative stress and reactive oxygen species, endoplasmic reticulum stress, calcium, nitrosative stress and *α*Synuclein Oligomerization.

## 1. Introduction

Parkinson's disease (PD) is a debilitating progressive neurodegenerative disorder for which there is no cure or long-term effective therapy. Classical symptoms include movement abnormalities such as postural instability and rigidity, tremors, and bradykinesia but more recently nonmotor symptoms have been ascribed to PD that include olfactory deficits, sleep disturbances, and gastrointestinal impairment. Although the molecular mechanisms underlying the disease are unknown, considerable evidence supports mitochondrial dysfunction and alpha-Synuclein (*α*Synuclein) as two of the major contributors to PD. This paper explores potential interrelationships between these two factors that may contribute to the initiation and/or progression of this disease.

## 2. Evidence for Dysfunctional Mitochondria Playing a Central Role in PD

Mitochondria are essential for neuronal function and survival. Energy-demanding neurons require large numbers of functional mitochondria to provide most of their ATP via oxidative phosphorylation (OXPHOS), a process where electrons traversing the electron transport chain (complexes I–IV) are coupled to proton pumping to establish a mitochondrial membrane potential subsequently used to synthesize ATP (complex V).

The involvement of mitochondrial dysfunction as a causal factor of PD is well supported by observations in patients [1, 2]. Impaired mitochondrial function is a predominant feature of PD with decreased activity in OXPHOS complexes and/or high levels of mitochondrial DNA deletions observed in PD-affected neurons [2]. In sporadic PD, altered or damaged mitochondrial proteins and impaired OXPHOS function are commonly observed in the region of the brain most damaged, the substantia nigra. Reduced levels of complex I subunits and impaired OXPHOS activity occur [3–5], with oxidative damage, functional impairment, and misassembly of complex I subunits found in PD brains [6]. Studies also observed mitochondrial complex I deficiency in other tissues of PD patients including muscle and platelets [7–9]. A stable decrease in complex I activity, increased ROS production, proton leak, and decreased maximum OXPHOS capacity also occur in PD cybrids (hybrid cells which contain the nuclear genome from a control and the mitochondrial genome from sporadic PD patients) [10, 11]. Furthermore, expression profiling of neurons from preclinical PD patients shows reduced mitochondrial biogenesis capacity [12]. All of these features are consistent with the involvement of OXPHOS dysfunction in sporadic PD, particularly with an emphasis on complex I. Important complementary evidence supporting this dysfunction as relevant to PD is derived from observations that complex I inhibitors cause Parkinsonism symptoms and neurodegeneration [13]. Rats continually exposed to rotenone, a complex I inhibitor, exhibit features of PD. Importantly, these rotenone-induced features were not due to the modest decrease in ATP levels resulting from rotenone treatment as an equivalent ATP loss-induced by 2-deoxyglucose was not toxic [14]. Furthermore, MPTP-derived MPP^+^, another complex I inhibitor, has been shown to cause Parkinsonism in humans and rodents [15].

Mutations in mitochondrial-associated proteins DJ-1, Parkin, and PINK1 also manifest themselves in familial forms of PD with a common theme involving defects in mitochondrial homeostasis, dynamics, and quality control. Mitochondria are the primary producers of reactive oxygen species (ROS), a byproduct of electron transfer in OXPHOS. Electrons may be leaked to oxygen, resulting in superoxide and other reactive oxygen species. A number of enzymatic antioxidants including superoxide dismutase (SOD1 cytosolic and SOD2 mitochondrial), catalase, and glutathione peroxidases exist to detoxify these harmful species [16]. Impairment of these antioxidant systems or an excess ROS generation results in oxidative stress that is well associated with PD. DJ-1 is a mitochondrial chaperone ascribed to providing neuroprotection against oxidative stress, and mutations in DJ-1 have been found to cause a form of autosomal recessive early-onset Parkinson's disease. Its role remains unclear, yet DJ-1 is reported to interact with complex I and may play a role in the maintenance of complex I activity [17]. This is consistent with its reduced expression, enhancing the toxicity caused by complex I inhibitors but not from agents that inhibit complexes II–V [18].

**Parkin and PINK1**

Mitochondria are remarkably dynamic organelles that constantly undergo a complex cycle of fusion and fission thought to be a crucial quality control system for the homeostasis of the mitochondrial network [19]. Fusion of mitochondria into a dynamic network allows the distribution and repair of mitochondrial proteins, mitochondrial genomic DNA, and lipids, whereas fission effectively isolates defective mitochondria from the network, which can then undergo a form of autophagic degradation, termed mitophagy [20]. Loss-of-function mutations in either Parkin or PINK1 can result in familial recessive forms of Parkinson's disease. PINK1 and Parkin act in concert to utilise the mitochondrial fission-fusion cycle to initiate the segregation and removal of irreversibly damaged/dysfunctional mitochondria with reduced mitochondrial membrane potential. Such mitochondria accumulate the putative serine/threonine kinase PINK1 on the outer mitochondrial membrane which in turn recruits the cytosolic ubiquitin ligase Parkin. Parkin-dependent ubiquitination/degradation of mitochondrially associated Miro results in mitochondria losing their attachment to microtubules and arresting their movement [21]. Fusion of the damaged mitochondria into the network is also prevented while the recruitment of autophagy components initiates the formation of a phagophore around the mitochondria prior to lysosomal delivery. That Parkin and PINK represent familial forms of Parkinson's disease clearly demonstrates the neuroprotective importance of efficiently disposing of damaged and dysfunctional mitochondria.

## 3. The Central Role of ***α***Synuclein in PD

Although the molecular mechanisms responsible for the disease are unknown, considerable evidence suggests the involvement of *α*Synuclein as a central component of PD. *α*Synuclein is the major structural constituent of cytoplasmic inclusion bodies (Lewy bodies) and neurites (Lewy neurites) that are characteristic of both familial and sporadic PD cases. Allele duplication or triplication of the wild-type *α*Synuclein gene (*SNCA*) results in autosomal dominant PD with a severity proportional to the degree of *α*Synuclein overexpression [22], and missense mutations in *α*Synuclein (e.g., A30P, A53T) are linked to dominantly inherited forms of PD [23]. Most significantly, genome wide association studies in multiple populations displayed that variation at the *SNCA* locus is robustly associated with sporadic PD [24–28].

*α*Synuclein is a small cytosolic protein capable of binding synaptic vesicles although its N-terminal lipid binding domain appears capable of binding to other membranes [29]. Its native function remains unclear and *α*Synuclein knockout mice show only a very mild phenotype [30]. With its dose-dependency and dominant inheritance pattern, *α*Synuclein is widely viewed as acting in a toxic manner with oligomeric species considered to mediate this toxicity instead of monomeric or the fibrillar forms found in Lewy bodies [31, 32]. Overexpressed and/or mutant *α*Synuclein has been ascribed to interfere with a number of intracellular processes that include membrane trafficking [33], autophagy [34], proteasomal degradation [35] and a range of mitochondrial functions [36].

## 4. An Interrelationship between ***α***Synuclein and Mitochondrial Dysfunction?

Recently *α*Synuclein has been reported to interact with mitochondria, raising the intriguing possibility that these two central components of PD might have a synergistic inter-relationship that contributes towards the complexities of PD. Additional evidence supporting such an interaction includes the following: (i) mice overexpressing *α*Synuclein treated with MPTP have significantly greater mitochondrial abnormalities than when treated with saline or wild-type mice treated with MPTP [37]; (ii) small molecules were identified that suppressed *α*Synuclein toxicity, and surprisingly they were found to also suppress the toxicity of a rotenone (complex I inhibitor) PD model [38].

Possible mechanisms of such an interrelationship include *α*Synuclein directly or indirectly impairing mitochondrial function, mitochondrial dysfunction exacerbating *α*Synuclein toxicity, or, perhaps more likely, *α*Synuclein toxicity and mitochondrial dysfunction enhancing each other in a self-amplifying cycle.

## 5. Potential Mechanisms by Which ***α***Synuclein Could Induce Mitochondrial Dysfunction

### 5.1. Does *α*Synuclein Physically Interact with Mitochondria?

*α*Synuclein has been detected in mitochondrial fractions from a range of model systems including cultured cells overexpressing *α*Synuclein [39] or mice brain homogenates [40]. Numerous immunohistochemistry studies have also identified *α*Synuclein association with mitochondria although some show association in regions of the brain not affected by PD. The questions regarding these findings have been as follows (i) does this indicate specific binding of *α*Synuclein to mitochondria or instead reflect a general membrane association of *α*Synuclein via its lipid binding domain, (ii) is *α*Synuclein associated with the cytosolic face of the outer mitochondrial membrane or has it been imported into the mitochondria, and (iii) does this mitochondrial association of *α*Synuclein contribute to the disease?

*In vitro*, *α*Synuclein appears to bind preferentially to highly curved, anionic lipid surfaces [41]. Mitochondria are resplendent in both curvature and anionic lipids such as cardiolipin. Indeed, liposomes derived from mitochondrial membranes, as well as liposomes rich in cardiolipin, bind *α*Synuclein while liposomes containing neutral lipids do not. Further, *α*Synuclein binding to cardiolipin is sensitive to competition from other cardiolipin-binding molecules, suggesting lipid-binding specificity [42].

Beyond simply binding to the surface of mitochondria, *α*Synuclein appears capable of being translocated across the outer mitochondrial membrane, likely by the outer mitochondrial translocation (TOM) machinery. Isolated mitochondria confer protease protection to recombinant *α*Synuclein, and this protection was lost in the presence of antibodies against TOM40. Additionally, both the OXPHOS uncoupler CCCP and the ATP synthase inhibitor oligomycin severely decreased *α*Synuclein import [43]. A cryptic mitochondrial targeting sequence has been suggested, and *α*Synuclein shares its lysine-rich N-terminus with a variety of nuclear-encoded mitochondrial proteins. These positive N-termini remain natively unfolded, like *α*Synuclein itself, potentially for recognition by mitochondrial import machinery [43, 44]. Although deletion of the highly positive N-terminus prevents the observed protease protection [43], it also removes the lysine-rich region that has been shown to confer specificity towards anionic lipids [41]. Therefore, it remains unclear whether the N-terminus leads to mitochondrial accumulation through direct membrane binding or through import machinery targeting.

*α*Synuclein specific localisation within mitochondria is less clear. Some immune-EM experiments show *α*Synuclein localised to the intermembrane space [43, 45], while others show distribution throughout both the intermembrane space and the matrix [46, 47]. The effort to elucidate *α*Synuclein location within mitochondria is complicated by the fact that translocation of *α*Synuclein to the matrix may, in fact, be a sign of mitochondrial dysfunction. It has been reported that cells transfected to overexpress *α*Synuclein had significant localisation of *α*Synuclein to the mitochondrial matrix, while vector-control-transfected cells showed only intermembrane localisation of endogenous *α*Synuclein. Mitochondria from *α*Synuclein overexpressing cells also showed significant morphological changes such as disorganised cristae, swelling of the intermembrane space, and gross fragmentation of both membranes [46]. It is possible that relocalisation of *α*Synuclein to the matrix may occur once significant damage has been done to the mitochondria, or once *α*Synuclein concentrations reach a threshold level. It is also possible that an unknown *α*Synuclein binding partner may promote or hinder translocation to the matrix, leading to disparate results across multiple disease models.

### 5.2. *α*Synuclein Translocates into Mitochondria and Impairs OXPHOS

The decrease in complex I activity, observed in patient brain samples [43, 48], transfected cell lines [43, 49], purified mitochondria [46], and transgenic animals [49, 50], is surprising in its specificity: none of the other OXPHOS complexes appear to be consistently affected by increased levels of *α*Synuclein [43, 48–50]. Perhaps most convincingly, complex I activity was negatively correlated with strong significance, with the amount of *α*Synuclein found within mitochondria isolated from patient brain samples [43]. Interestingly, the inhibition of complex I was only observed within the substantia nigra (SN), and no correlation between *α*Synuclein levels and complex I activity was observed in the cerebellum. However, mitochondrial *α*Synuclein expression was significantly lower in the cerebellum (roughly tenfold less) and perhaps had not reached toxic threshold levels [43]. This data suggests that *α*Synuclein could be integral to the observed loss of complex I activity, but that reaching toxic intramitochondrial levels may require additional tissue-specific factors that influence import of the protein.

 The intermembrane space localisation of *α*Synuclein leads to the possibility of a direct interaction between *α*Synuclein and complex I. Indeed, in patient brain samples examined by blue native PAGE *α*Synuclein was found to localise to bands representing complex I. Furthermore, in patient samples and transfected cultured cells, but not healthy controls or transfected cells grown for a shorter time, multiple smaller bands positive for a complex I subunit were observed, suggesting a deficiency in the assembly of the complex I holoenzyme [43]. It is important to note that while other researchers have noted an impairment in complex I activity in transgenic mice with no impairment of assembly, these researchers did not examine cross-reactivity to *α*Synuclein [49], and therefore a direct interaction cannot be eliminated in these mice models of PD.

What form of *α*Synuclein might be responsible for the observed complex I inhibition? Induction of high-molecular-weight *α*Synuclein oligomers in cultured dopaminergic cells via exposure to 18 : 3 polyunsaturated fat [51] did not inhibit complex I activity in these cells [49]. Additionally, transgenic mice expressing the A53T mutant form of *α*Synuclein showed no correlation between levels of aggregated *α*Synuclein, measured by immunoblot, and complex 1 activity [49]. It is possible that monomeric *α*Synuclein and oligomeric *α*Synuclein are toxic to cells in disparate ways, the former hampering OXPHOS function while the latter leads to deficiencies in transport, autophagy, and protein degradation. Certainly, impairment of the OXPHOS could lead to dissipated membrane potential and lower levels of oxidative phosphorylation. This could alter the available ATP, exacerbating stresses placed on the cell by oligomeric inclusions of *α*Synuclein. These cellular stresses could, in turn, increase mitochondrial stress via Ca^2+^ intake, leading to a cycle of increasing dysfunction in various subcellular locations.

As with determining mitochondrial localisation of *α*Synuclein, there are conflicting reports as to how *α*Synuclein affects membrane potential and ATP production. *In vitro*, recombinant *α*Synuclein lowers membrane potential and ATP production in isolated mitochondria without any discernable effects on the activity of the OXPHOS complexes. *α*Synuclein concentrations as low as double endogenous concentrations were sufficient to observe loss of membrane potential and ATP production [52]. *In vivo*, loss of membrane potential, visualised by TMRM fluorescence, and O_2_ consumption were both observed in transfected cells overexpressing *α*Synuclein. Consistent with the severe pathology associated with A53T mutations in *α*Synuclein, the effects on membrane potential and O_2_ consumption were more severe when the mutant protein was expressed [53]. However, other mitochondrial pathologies, such as increased fragmentation and decreased rate of fusion, have been reported in the absence of impairment of respiration or loss of membrane potential [45]. This raises the question: could *α*Synuclein be acting at multiple locations on and within mitochondria to influence different functions through disparate physical interactions? or is there a central factor influenced by *α*Synuclein that, in turn, influences a variety of mitochondrial functions?

One crucial family of molecules within the mitochondria are the cardiolipin family of phospholipids. These fatty acids are unusual in their bicyclic nature, yielding a phospholipid with four, rather than two, fatty acid tails. Cardiolipins are implicated in a variety of critical processes such as mitochondrial fusion, protein complex stability, and metabolite transport [54]. Loss of cardiolipin in *C. elegans* (via knockout of a gene required for cardiolipin synthesis) leads to the loss of mitochondrial membrane potential and distortion of cristae within germ cells [55], and similar results were observed in *S. cerevisiae* [56]. Even more directly, *in vitro *experiments on purified OXPHOS complexes showed complex I to be dependent on cardiolipin for proper electron transfer [57]. In summary, cardiolipin is integral to many mitochondrial processes found to be deficient in models of PD.

 Cardiolipin therefore proves to be an interesting target for *α*Synuclein toxicity. Its known involvement in PD impaired processes, as well as observed specific binding of cardiolipin by *α*Synuclein points towards an important molecular interaction. Somewhat surprisingly, *α*Synuclein-knockout mice show a decrease in mitochondrial cardiolipin, cardiolipin precursors, and complex I-/III-linked activity (electron transfer from complex I to complex III) yet no impairment of any single complex. Due to the mitochondrial localisation of cardiolipin synthesis and the ability of *α*Synuclein to bind cardiolipin and its precursors, the authors suggest that endogenous *α*Synuclein may in fact help target lipids to the mitochondria for use in other biosynthetic pathways [58]. Conceivably, tight binding of cardiolipin by *α*Synuclein could prevent proper function. This impairment would be a proportional interaction, where higher levels of *α*Synuclein would deplete the bioavailable cardiolipin pool in a one to one ratio, consistent with the observation that complex I inhibition is proportional to *α*Synuclein levels [43]. Unfortunately, there is a lack of literature on the effects of high *α*Synuclein levels on cardiolipin. However, due to the well-documented interactions between cardiolipin and *α*Synuclein, and the similarity between pathologies caused by loss of cardiolipin and overexpression of *α*Synuclein, further investigation into their interrelationship seems well warranted.

### 5.3. *α*Synuclein Perturbs the Mitochondrial Fission-Fusion Cycle

*α*Synuclein lipid-binding capacity and proposed specificity for mitochondrial membranes resulted in several recent studies focused on the involvement of *α*Synuclein in inhibiting the crucial and delicately balanced mitochondrial fission-fusion cycle [59]. Mitochondrial fusion is an essential process of mitochondrial dynamics, involved in the maintenance of mitochondrial homeostasis. Mitochondrial fusion maintains proper OXPHOS functionality by promoting the sharing of integral proteins across the mitochondrial population. Fusion is also required for the proper segregation and inheritance of mitochondrial DNA. Mitochondrial fusion enables the exchange of contents between mitochondria and allows damaged mitochondria to acquire components from healthy mitochondria, diluting damage that would otherwise require disposal of an individual mitochondrion [20].

Elevated *α*Synuclein expression in cultured cells resulted in *α*Synuclein binding mitochondria and mitochondrial fragmentation whereas siRNA-mediated knockdown of *α*Synuclein resulted in elongated mitochondria [45]. Such observations could be due to *α*Synuclein either inhibiting mitochondrial fusion or enhancing mitochondrial fission or alternatively impairing OXPHOS that in turn results in fragmentation.

#### 5.3.1. *α*Synuclein Inhibits Mitochondrial Fusion

Evidence supporting *α*Synuclein as an inhibitor of mitochondrial fusion utilised *in vitro* fusion experiments where fusion of small artificial unilamellar vesicles was suppressed by recombinant *α*Synuclein in a dose-dependent manner [45]. The overexpression of *α*Synuclein in neuroblastoma cells was also found to severely decrease mitochondrial fusion events. Cells expressing either GFP- or DsRed-tagged mitochondria were artificially fused via PEG treatment and mitochondrial fusion monitored by DsRed colocalization [45]. Promoting lengthened, tubular mitochondria via the elevated expression of fusion-promoting proteins Mfn1, Mfn2 and Opa1, or alternatively via the knockdown of the fission-promoting protein Drp1 was found to reduce the proportion of fragmented mitochondria in cells challenged with *α*Synuclein. However, the overexpression of fusion proteins was not sufficient to rescue mitochondrial morphology. Additionally both Drp1 knockdowns and cells expressing dominant-negative Drp1 still showed decreased mitochondrial length upon *α*Synuclein expression. This led the authors to conclude that *α*Synuclein inhibition of fusion acted independently of mitochondrial fusion and fission proteins [45, 47]. Even endogenous levels of *α*Synuclein appear to impede mitochondrial fusion. Having induced mitochondrial fragmentation with CCCP, an ionophore which dissipates mitochondrial membrane potential, the fusion-dependent morphologic recovery after CCCP washout was slower in cells expressing endogenous *α*Synuclein levels compared to *α*Synuclein siRNA knockdown cells [45]. *α*Synuclein was proposed to function as a stabiliser of lipid packing defects in highly curved membranes. Essentially acting as a bandage, *α*Synuclein “patches” itself on the membrane at an area of high curvature, where a fusion event would normally occur, effectively halting the fusion process [45]. This model agrees with data showing *α*Synuclein preferentially binding to areas of high membrane curvature [41]. Further supporting the possibility of *α*Synuclein sterically hindering fusion is the fact that cardiolipin, a strong binding partner for *α*Synuclein, is often enriched in fusion event zones.

#### 5.3.2. *α*Synuclein Induces Mitochondrial Fragmentation

In contrast, other studies have found that *α*Synuclein does not impair mitochondrial fusion but instead is capable of enhancing mitochondrial fragmentation [47]. The mitochondrial fission promoting properties of *α*Synuclein were independent of the native fission machinery and required the membrane-binding N-terminus of *α*Synuclein [47], suggesting that *α*Synuclein may fragment mitochondria by directly binding and disrupting mitochondrial membranes. *In vitro, *cardiolipin-containing vesicles, but not vesicles lacking cardiolipin, were found to decrease in size when exposed to recombinant *α*Synuclein [47]. Further analysis supported the assertion that oligomeric *α*Synuclein was responsible for the fission observed which, given that monomeric *α*Synuclein can also bind membranes, suggests binding alone is insufficient [47].

It is clear that *α*Synuclein causes mitochondrial fragmentation, but it is unclear if this is a result of increased fission, impaired fusion or a combination of both.

### 5.4. *α*Synuclein Inhibition of Autophagy and Mitophagy

Mitophagy is a specialised form of autophagy to remove and dispose of dysfunctional mitochondria. Mitophagy uses componentns of the autophagy machinery to encapsulate the mitochondrion in a phagophore membrane and deliver it to the lysosome for degradation. The ability of *α*Synuclein to impair macroautophagy [60] would have the potential to impair mitophagy, thereby causing an accumulation of dysfunctional and potentially ROS producing mitochondria. Interestingly, neither the *α*Synuclein mutants A30P nor A53T were found to impair macroautophagy [60].

In contrast to *α*Synuclein impairing autophagy, Choubey et al. propose that expression of *α*Synuclein mutant A53T might in fact induce detrimental supraphysiological levels of mitophagy/autophagy. The elevated expression of mutant A53T *α*Synuclein in primary neurons resulted in extensive mitochondrial loss resulting in a bioenergetic deficit [61]. The mitochondrial loss required mitochondrial fragmentation, Parkin, and the autophagy protein Beclin, suggesting mitophagy was responsible. The authors proposed that A53T *α*Synuclein had caused an overactivation of autophagy that resulted in the excessive disposal of functional polarised mitochondria. This is consistent with EM analysis of mouse midbrain dopaminergic neurons expressing high levels of mutant A53T *α*Synuclein which displayed an increase in the proportion of mitochondria sequestered in double-membraned structures, presumably autophagosomes [50]. However, an alternative possibility is that *α*Synuclein impaired the later membrane trafficking stages of autophagy/mitophagy involving fusion with lysosomes, which would cause an accumulation of autophagosomes. Choubey et al. utilised a pH-sensitive dual-fluor LC3 construct to explore the rate of autophagosome delivery to lysosomes, and found no change in the presence of *α*Synuclein [61]. However, multivesicular bodies (MVBs), another destination for autophagosomes [62], are also acidic [63] and delivery to an MVB would likely cause the LC3 construct to behave similarly, as if delivered to a lysosome. Therefore, a trafficking impairment to lysosomes cannot be dismissed. Further, it is unclear if the induction of mitophagy is initiated at mitochondria themselves, or whether the observed increase in mitophagy is a secondary effect of general upregulation of macroautophagy. Knockdowns of both Parkin, crucial for induction of mitophagy at the mitochondrial level, and Beclin, involved in assembly of the autophagophore, lead to similar increases in survival of *α*Synuclein-challenged cultured neurons.

### 5.5. *α*Synuclein-Induced Endoplasmic Reticulum (ER) Stress Impairs Mitochondrial Function

The endoplasmic reticulum (ER) is a multifunctional organelle essential for cellular processes including lipid synthesis, regulation of calcium homeostasis, and biosynthesis of proteins destined for intracellular organelles, the cell surface, or secretion [64]. Intriguingly the ER is intimately connected, both physically and functionally, to the mitochondria allowing the signalling and exchange of metabolites between these two organelles [65], including phospholipid exchange for biosynthesis and controlled release of Ca^2+^ from the ER to the mitochondria [66, 67]. To accomplish this, a multiprotein complex tethers ER membranes to mitochondrial outer membranes in close association in a structure termed MAM (mitochondria-associated ER membrane) [65, 66]. Recently the ER has also been shown to provide a role in mitochondrial fission. In both yeast and mammalian ER-mitochondrial contact sites ER tubules constrict mitochondrial tubules to a size sufficiently small enough for the DRP1 ring-like structure to perform its scission function during mitochondrial fission [68]. The close physical and functional association between the ER and mitochondria provides the opportunity for stress or dysfunction in one organelle to potentially disrupt homeostasis in the other organelle. ER stress has been detected in dopaminergic neurons of the substantia nigra bearing *α*Synuclein inclusions in the brain of patients affected by PD, indicating that ER stress is involved in sporadic PD [69]. Furthermore the ER stress observed closely correlated with the accumulation and aggregation of *α*Synuclein [69]. Elevated *α*Synuclein levels have been found to block ER to Golgi membrane trafficking [33, 70] and cause ER stress [33], raising the possibility that *α*Synuclein-induced ER stress might play a role in precipitating mitochondrial stress [71, 72].

The ER is the main cellular calcium store, but under conditions of prolonged ER stress, the ER releases calcium into the cytosol, resulting in the transfer of calcium to mitochondria which effectively buffers cytosolic calcium levels [73]. However, the resulting mitochondrial calcium levels, if too high, can lead to a depolarisation of mitochondrial membrane potential, decreased ATP synthesis, and ultimately the opening of the permeability transition pore leading to mitochondrial swelling and eventually death [73]. Interestingly, MAM sites appear to be dependent on MFN2 (a mitochondrial fusion promoting protein), as loss of this protein results in increased ER-mitochondrial distances and ablation of ER-mitochondrial calcium transfer [73]. As discussed earlier, MFN2 can suppress *α*Synuclein-induced mitochondrial morphological changes (though not fully reverse them) in cultured cells [45] as well as decrease autophagosome-associated mitochondria in neurons challenged with the A53T mutant of *α*Synuclein [61]. The ability of MFN2 to regulate ER-mitochondrial distance and mediate calcium uptake could explain the cytotoxicity in MFN2 overexpressing cultured neurons reported by Choubey et al. due to the mitotoxic effect of chronic high mitochondrial calcium levels [61]. Further, ER structure is dependent on MFN2, indicating a reciprocal relationship between the ER and mitochondria [73] and reinforcing the possibility of an escalating cycle of toxicity between the two organelles.

ER stress might also negatively impact mitochondria by perturbing lipid biosynthesis. The synthesis of lipids such as cardiolipin is initiated in the ER but completed in the mitochondria, requiring the exchange of lipid intermediates between the ER membrane and the mitochondrial outer membrane at contact sites [54]. ER stress might impair the synthesis of the intermediates within the ER or disturb contact points required for the exchange of lipid intermediates, potentially leaving the mitochondria deficient in cardiolipin which would result in reduced OXPHOS efficiency [74].

 Finally loss of ER homeostasis could impact the contribution of the ER in mitochondrial fission, a process important in mitochondrial homeostasis, especially for the efficient removal of damaged mitochondria by mitophagy. Therefore *α*Synuclein-induced ER stress could induce mitochondrial dysfunction in a myriad of ways.

### 5.6. *α*Synuclein May Interfere with the Transport/Distribution/Disposal of Mitochondria

In the long processes of neurons, appropriate transport and distribution of mitochondria is critical to supply both the required high metabolic demands as well as to sequester excess intracellular Ca^2+^ to maintain Ca^2+^ homeostasis at synapses. In addition to the anterograde delivery of functional mitochondria, retrograde transport is required for delivering dysfunctional mitochondria to the lysosome for mitophagic degradation. Long-range mitochondrial transport between the soma and distal axonal and dendritic regions is reliant on microtubules and associated motors [75]. Perturbation of microtubule transport would impair both the appropriate delivery and distribution of mitochondria within neurons, particularly from the critical area of the synaptic terminals, as well as the disposal of potentially ROS producing dysfunctional mitochondria.

Microtubules are dynamic structures, and *α*Synuclein may play a role in modulating the polymerisation and depolymerisation of tubulin. Recombinant GST-*α*Synuclein was found to bind tubulin *in vitro* while the transient expression of *α*Synuclein in HeLa cells was reported to disrupt microtubule structures [76]. Furthermore, a dopaminergic cell line exposed to extracellular oligomeric *α*Synuclein exhibited decreased tubulin polymerisation. At a concentration of 250nM, neither monomeric nor oligomeric *α*Synuclein affected tubulin polymerisation *in vitro. *[77]. However, higher molar ratios of *α*Synuclein to tubulin lead to a significant increase in polymerisation rate, measured by OD [76], [78]. Strengthening the case for a direct interaction between *α*Synuclein and tubulin are multiple co-IP and *in vitro* polymerisation experiments utilising *α*Synuclein-deletion mutants. These experiments indicate that only amino acids 61–100 of *α*Synuclein are required for observed enhancement of polymerisation. Additionally, tubulin has been shown to dramatically increase the rate of *α*Synuclein fibrilization, measured by Thio-T fluorescence, suggesting a possible seeding of these fibrils [80]. Contrary to this apparent enhancement, cells exposed to extracellular oligomeric *α*Synuclein and cybrids formed with mitochondria from PD patients both showed increased ratios of free tubulin to polymerised tubulin, suggesting an inhibition of polymerisation [10, 77]. Surprisingly, treatment of these cybrids with taxol, an enhancer of tubulin polymerisation, restored the free/polymerised tubulin ratio to control levels in PD cybrids with no discernable effect on control cybrids [10]. Additionally, taxol treatment eliminated high-molecular-weight *α*Synuclein oligomers observed in PD, but not control, cybrids. Also of note, both the A30P and A53T mutant forms of *α*Synuclein appeared to influence the structure of polymerised tubulin, and the presence of either mutant *in vitro* led to amorphous polymers lacking tubular structure [78]. While the precise mechanism by which *α*Synuclein may influence polymerisation or improper aggregation of tubulin is not clear, it is apparent that an interaction between the two proteins exists. Considering the importance of microtubule tracks for proper distribution of mitochondria and intracellular transport, it is clear that perturbation of the microtubule network could significantly impact the mitochondrial fission/fusion cycle and the mitophagy of ROS-producing mitochondria or lead to ER stress.

## 6. Potential Mechanisms by Which Mitochondrial Dysfunction Could Enhance ***α***Synuclein Toxicity

### 6.1. *α*Synuclein Upregulation by Oxidative Stress Resulting from Dysfunctional Mitochondria

ROS-producing dysfunctional mitochondria may impact *α*Synuclein in several ways. The resulting oxidative stress from dysfunctional mitochondrial could cause upregulation of *α*Synuclein expression [81]. Given that *α*Synuclein toxicity is dose dependent, any increase in *α*Synuclein expression would be expected to increase toxicity.

### 6.2. ER Stress Induced by Mitochondrial Dysfunction

Given the physically and functionally intimate relationship between the ER and mitochondria, mitochondrial dysfunction could cause ER stress, enhancing ER stress induced by *α*Synuclein [33, 70]. This concept is experimentally supported with compounds that inhibit OXPHOS found to cause ER stress. The compounds MPP+ and rotenone are both used to reproduce PD features in model organisms, and in addition to being mitochondrial complex I inhibitors, they induce ER stress in cultured neuronal cells [82], [79]. Inhibition of complex III with antimycin or complex V (ATPase synthetase) with oligomycin also resulted in ER stress as judged by the upregulation of ER stress response genes [83, 84]. This induction of the ER stress response is likely due to a disturbance in mitochondrial Ca^2+^ buffering as either Ca^2+^ chelation with BAPTA or preventing Ca^2+^ release from the oligomycin-treated mitochondria significantly suppressed the ER stress [83]. Mitochondrial dysfunction can trigger a release of mitochondrial Ca^2+^ that in turn triggers ER stress. As the ER is the main store of Ca^2+^ within the cell, ER stress results in a further release of Ca^2+^. Therefore mitochondrial dysfunction can cause a release of Ca^2+^ from the cell's two largest Ca^2+^ storage organelles, the mitochondria, and ER. The resulting significant increase in cytosolic Ca^2+^ levels, derived from both the mitochondria and ER, can in turn promote *α*Synuclein oligomerisation/aggregation [85]. This effect on *α*Synuclein could potentially create a vicious circle that would lead to increased mitochondrial dysfunction and/or increased *α*Synuclein-dependent ER stress. Finally, ER stress induced by mitochondrial dysfunction might in turn impair lipid biosynthesis occurring in the ER, enhancing sensitivity to toxic species of *α*Synuclein [86].

### 6.3. Mitochondrial Dysfunction Induced Nitric Oxide Production and Nitrosative Stress

Mitochondrial dysfunction or ER stress could elevate cytosolic calcium to levels capable of activating neuronal nitric oxide synthetase (nNOS). The resulting nitric oxide (NO) can react with superoxide to produce the toxic species *peroxynitrite*.* Peroxynitrite* can oxidatively modify proteins, lipids, and nucleic acids resulting in a range of damages including proteasome inhibition, ER stress, and mitochondrial damage including inhibition of complex I and a decrease in mitochondrial membrane potential [87]. NO and peroxynitrite can decrease glutathione levels, increasing the cell vulnerability to oxidative stress, but can also cause either tyrosine nitration or nitrosylation of cysteine residues, often altering the protein's function/activity. In PD, proteins with abnormal S-nitrosylation include Parkin (a ubiquitin ligase and a familial PD gene), protein disulfide isomerase (PDI, whose impairment causes ER stress), Prx2 (a protein that protects against oxidative stress in neurons but is inactivated by S-nitrosylation) [88], and *α*Synuclein, with Lewy bodies enriched with the nitrated species [89, 90]. Finally, polymorphisms in neuronal NOS (nNOS) and inducible NOS (iNOS) are both associated with sporadic PD [91].

The initiation of autophagy can be impaired by nitric oxide [92] which raises the possibility that mitochondrial dysfunction could impair autophagy via the induction of NOS and resulting nitric oxide production. Impairment of autophagy could in turn impair both the mitophagic disposal of dysfunctional mitochondria as well as the autophagic removal of *α*Synuclein.

### 6.4. ROS from Mitochondrial Dysfunction Enhances/Stabilises *α*Synuclein Oligomerisation and Increases *α*Synuclein Toxicity

Increases in ROS can also lead to modifications of *α*Synuclein itself. High levels of ROS in the presence of nitric oxide can lead to the production of reactive nitrogen species (RNS). Both ROS and RNS can directly modify *α*Synuclein at crucial residues; however, RNS may play an important role in *α*Synuclein oligomerisation and aggregation. RNS can directly nitrate tyrosine residues, altering *α*Synuclein local hydrophobicity and charge. *α*Synuclein has four tyrosine residues, one in the lipid-binding and lysine-rich region of the protein, tyr_1_, and three clustered in the C-terminal acidic region, tyr_2−4_ [93]. Modification of these residues significantly alters both the native conformation of the protein and its lipid binding dynamics. Nitration at either tyr_1_ or tyr_2-4_ leads to a drastic (~50%) decrease in binding to small, anionic vesicles. Interestingly, the A30P mutation shows a similar degree of membrane affinity loss. This effect could be mimicked for tyr_1_ through a tyrosine to aspartic acid targeted mutation, suggesting an electrostatic basis for the loss of binding. However, similar mutations to tyr_2-4_ did not alter membrane binding, indicating a long distance, allosteric basis for effect of C-terminal nitration on membrane binding [93].

 However, nitration does not simply alter the membrane binding properties of *α*Synuclein. Nitration also alters the way in which *α*Synuclein oligomerises. Native *α*Synuclein can easily be induced to form long fibrils via agitation, incubation at physiological temperatures, and time. However, nitrated *α*Synuclein does not form fibrils unless incubated at pH 3. Additionally, the presence of the nitrated species significantly slowed the fibrillation of unmodified *α*Synuclein [93]. While nitration prevented fibrillation, it accelerated the formation of stabilised oligomers [94]. One proposed mechanism for stabilisation of oligomeric, modified *α*Synuclein is RNS-induced dityrosine crosslinking. Crosslinks formed by exposure to peroxynitrate, an RNS, are extremely stable, remaining intact even after treatment with 4 M urea. Consistent with earlier findings, crosslinked *α*Synuclein does not form fibrils. However, fully formed fibrils exposed to RNS become likewise crosslinked and resistant to denaturants [95]. It is likely that the nitrated *α*Synuclein detected in intracellular inclusions [93, 94] was modified after deposition, considering the resistance to fibrillation exhibited by nitrated *α*Synuclein.

 However, it is still not clear how prevention of fibril development and stabilisation of small, soluble oligomers might induce toxicity. One possibility is through the impairment of chaperone-mediated autophagy (CMA) at lysosomes. In isolated lysosomes nitrated *α*Synuclein was significantly impaired in translocating the lysosomal membrane [34]. Accumulation of *α*Synuclein at the surface of lysosomes could have deleterious effects on the fusion of autophagosomes, providing a possible explanation for the perturbations in the autophagy pathway discussed earlier, as well as preventing normal CMA-mediated protein turnover.

## 7. Summary

**Figure 1:**
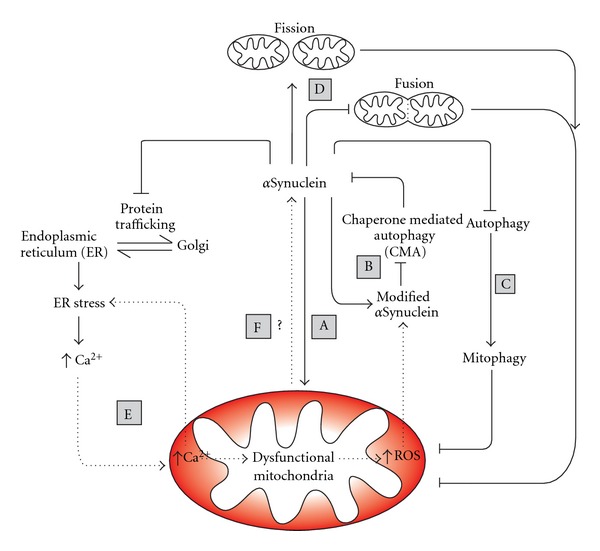
The interrelationship between mitochondrial dysfunction and *α*Synuclein toxicity. *α*Synuclein toxicity, directly or indirectly, impairs mitochondrial function (A). A prominent result of this dysfunction is the production of reactive oxygen species (ROS) which can be counteracted by the cellular ROS buffering systems. However prolonged mitochondrial stress, exacerbated by *α*Synuclein, has the potential to deplete this buffering capacity, and the resulting increase of cellular ROS has multiple damaging effects such as the modification of *α*Synuclein (B). Modified *α*Synuclein can inhibit chaperone-mediated autophagy (CMA), increasing the proteins toxicity by its inefficient clearance. *α*Synuclein toxicity is dose dependant, and excessive amounts of *α*Synuclein have the potential to block autophagy pathways (i.e., macroautophagy and mitophagy). This results in an accumulation of dysfunctional mitochondria due to inefficient clearance (C). *α*Synuclein toxicity can also increase mitochondrial fission and inhibit mitochondrial fusion (D). Both the increase in mitochondrial fragmentation and the inability of mitochondria to rejoin the mitochondrial network result in an increase in dysfunctional, depolarised mitochondria. *α*Synuclein toxicity also blocks endoplasmic reticulum (ER) to Golgi trafficking resulting in ER stress. When under constant and prolonged stress, the ER releases Ca^2+^ into the cytosol. Due to mitochondrial-ER contact sites, mitochondria readily buffer cytosolic Ca^2+^; however, excess Ca^2+^ in the mitochondria causes mitochondrial stress (E). Dysfunctional mitochondria in turn release Ca^2+^ into the cytosol causing further ER stress. Mitochondrial dysfunction may exacerbate *α*Synuclein toxicity (F), with both acting synergistically to enhance each other in a self-amplifying cycle over prolonged periods of time, resulting in multiple downstream effects, including cell death as seen in PD.

It is clear that numerous potential mechanisms exist in which mitochondrial dysfunction and *α*Synuclein could interact to exacerbate their neurodegenerative properties (see Figure 1). In many cases a self-amplifying cycle of mitochondrial dysfunction or *α*Synuclein toxicity could be envisaged as either being the disease-initiating factor yet acting together during disease progression.

It is important to acknowledge that most in the PD field view mitochondrial dysfunction through the lens of OXPHOS impairment and increased ROS production, yet the mitochondria contribute to so many other facets of cell metabolism including, TCA cycle, NAD^+^/NADP, and amino acid biosynthesis, as well as its close relationship in the function and maintenance of other cellular organelles, with perturbations in many of these processes potentially also contributing to PD.
